# MiRNA-based “fitness score” to assess the individual response to diet, metabolism, and exercise

**DOI:** 10.1080/15502783.2022.2106148

**Published:** 2022-08-02

**Authors:** Ulrike D.B. Krammer, Sylvia Tschida, Julia Berner, Stephanie Lilja, Olivier J. Switzeny, Berit Hippe, Petra Rust, Alexander G. Haslberger

**Affiliations:** a Department of Nutritional Sciences, University of Vienna, Vienna, Austria; b HealthBioCare GmbH, Vienna, Austria

**Keywords:** Exercise, epigenetic, miRNAs, biomarker, fitness score, nutrition

## Abstract

**Background:**

Regular, especially sustained exercise plays an important role in the prevention and treatment of multiple chronic diseases. Some of the underlying molecular and cellular mechanisms behind the adaptive response to physical activity are still unclear, but recent findings suggest a possible role of epigenetic mechanisms, especially miRNAs, in the progression and management of exercise-related changes. Due to the combination of the analysis of epigenetic biomarkers (miRNAs), the intake of food and supplements, and genetic dispositions, a “fitness score” was evaluated to assess the individual response to nutrition, exercise, and metabolic influence.

**Methods:**

In response to a 12-week sports intervention, we analyzed genetic and epigenetic biomarkers in capillary blood from 61 sedentary, healthy participants (66.1% females, 33.9% males, mean age 33 years), including *Line-1* methylation, three SNPs, and ten miRNAs using HRM and qPCR analysis. These biomarkers were also analyzed in a healthy, age- and sex-matched control group (n, 20) without intervention. Food frequency intake, including dietary supplement intake, and general health questionnaires were surveyed under the supervision of trained staff.

**Results:**

Exercise training decreased the expression of miR-20a-5p, −22-5p, and −505-3p (p < 0.02) and improved the “fitness score,” which estimates eight different lifestyle factors to assess, nutrition, inflammation, cardiovascular fitness, injury risk, regeneration, muscle and hydration status, as well as stress level. In addition, we were able to determine correlations between individual miRNAs, miR-20a-5p, −22-5p, and −101-3p (p < 0.04), and the genetic predisposition for endurance and/or strength and obesity risk (*ACE, ACTN3*, and *FTO*), as well as between miRNAs and the body composition (p < 0.05). MiR-19b-3p and −101-3p correlated with the intake of B vitamins. Further, miR-19b-3p correlated with magnesium and miR-378a-3p with iron intake (p < 0.05).

**Conclusions:**

In summary, our results indicate that a combined analysis of several biomarkers (miRNAs) can provide information about an individual’s training adaptions/fitness, body composition, nutritional needs, and possible recovery. In contrast to most studies using muscle biopsies, we were able to show that these biomarkers can also be measured using a minimally invasive method.

## Introduction

Regular, especially sustained, exercise plays an important role in the prevention and treatment of multiple chronic diseases, such as cardiovascular diseases, metabolic disorders, neurological/cognitive diseases, musculoskeletal disorders, and cancer, but also for the maintenance of health and well-being [1]. In addition, aging conditions, such as a decline in cognitive/motor functions, and loss of muscle mass and strength, can be improved through regular exercise and age-related deterioration can be delayed [2,3]. Moreover, our genetic architecture and epigenetic manifestations play an essential role in this big area, for example, adherence to exercise, a person’s response to exercise, and metabolic adaptions through exercise [4].

Variations in voluntary physical activity are an important determinant of long-term human health. The predisposition to voluntary activity is heritable and leads to protective metabolic changes [5]. From animal and human research, however, it is known that neural signaling and pleasure/reward systems in the brain to a large extent promote the tendency to be physically active and to adhere to a training program. Based on a large epidemiologic study (TIGER study), 26 single nucleotide polymorphisms (SNPs) in six candidate genes (*BDNF, BDNFOS, DRD2, DRD4, HTR2A*, and *HTR2C*) were identified that are related to behavior during physical activity, that is, compliance, duration, intensity, and total dose of exercise in young men and women [6]. On the other hand, it is assumed that there is also a genetic predisposition to the type of sport (endurance *ACTN3* or strength *ACE*) a human is better at. One example is the *Alpha-actinin-3* (*ACTN3*) genotype, which affects human performance in various ways through the absence or presence of α-actinin-3 in skeletal muscle [7]. In addition, variants of the *Fat mass- and obesity-associated* (*FTO*) gene show a strong association with obesity and fat mass but are also described to influence the skeletal muscle phenotypes of athletes [8]. A study by Almén et al., 2012 [9] suggests that the effect of the *FTO* obesity risk allele may be mediated through epigenetic changes.

On the other hand, genetic dispositions alone are insufficient to explain the individual response to diet, nutrition, and exercise. It has already been shown that exercise can alter gene expression [10] and that an early response to physical activity triggers increased transcription of regulatory, metabolic, and myogenic genes, which are important for mediating subsequent adaptations in skeletal muscle and contribute to improved fitness [4]. Through increased transcription, exercise can change the methylation status of certain genes that are involved in muscle function. And this can lead to the formation of a long-lasting favorable expression pattern for improved training ability. In response to physical activity, the methylation profile changes in a dose-dependent, gene-specific, and tissue-specific manner [11,12]. MiRNAs are small (approx. 22 nucleotides in length), highly conserved noncoding RNA molecules, which are also altered by physical activity [13] and they can influence gene expression post-transcriptional. MiRNAs bind to their miRNA response elements (MREs) situated on RNAs and upon binding initiate the degradation of the RNA and inhibit their translation, that is, miRNAs silence-specific genes, so the expression of miRNAs depends on the exercise type, duration, intensity, and tissue. Therefore, miRNAs are subdivided into cardiac-, and muscle depending, and circulated miRNAs (ci-miRNAs) [14,15].

In addition, to understand the various pathways that physical activity triggers in the human body, there is growing interest and the use of so-called biomarkers, which individually reflect and evaluate the health, performance, and recovery of every human. This information could help athletes and/or coaches to improve performance while reducing the risk of overtraining and injury. At the moment, mainly proteins or electrolytes are used as biomarkers, which are measured in blood, urine, or saliva, but also genetic tests [16]. By the time, these markers are detectable in the circulation; however, consequential damage, such as inflammation and injuries may already have occurred. It is assumed that changes in miRNA expressions in body fluids will occur earlier than with conventional biomarkers, which is why miRNAs are more favorable for the early detection of possible deficits [17].

The main goal of our work was to identify miRNAs that are influenced by endurance and strength training, and are suitable as biomarkers to provide information about the current fitness and health status of a person. This can even be achieved through a noninvasive method using capillary blood [13], which makes it easier for athletes and trainers to take samples and counteract possible deficits immediately. Additionally, there is increasing evidence that finger capillary blood sampling can provide a reliable alternative to venous blood sampling in clinical and field settings [18]. Furthermore, another aim was to identify possible interactions between selected epigenetic and genetic markers, the diet, and the anthropometric data to be able to make personal recommendations for athletes in the future. Since the literature almost exclusively contains sports intervention studies with men, with this study we also wanted to create a certain basis for the interactions and effects of exercise and epigenetics in women.

## Materials and methods

### Marker selection

**Figure 1.**
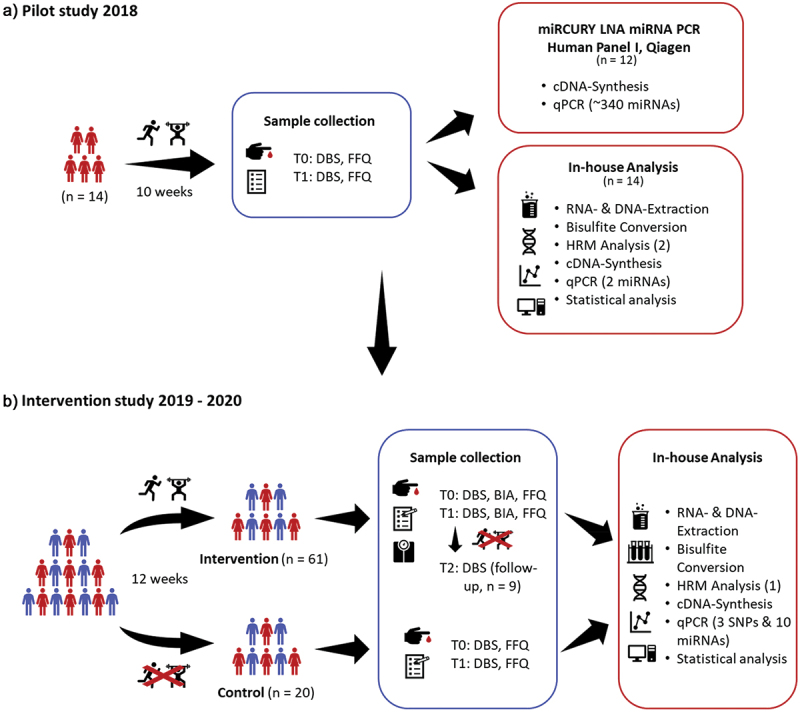
Study design of the pilot study 2018 (**A**) and the intervention study 2019 − 2020 (**B**). T0, begin, T1, after 10-/12-weeks, T2, after 10-month, DBS, dried blood spot, BIA, bioelectrical impedance analysis, FFQ, food frequency questionnaire, HRM, high-resolution melting, SNP, single nucleotide polymorphism.

In a pilot study from 2018, we analyzed over 340 different miRNAs and two methylation sites from capillary blood from 14 healthy women, aged between 36 and 59 years, with an average BMI of 32 kg/m^2^. The results of this study as well as literature data were used to set up and select the markers for the intervention of the following sport study (Figure 1 A and B).

### Study design of the sports intervention study

For the sports intervention study at the University of Vienna, Department of Nutritional Science, over 90 participants, women, and men, aged between 19 and 55 years, who were physically inactive, and had a BMI between 20 and 35 kg/m^2^ (Table S1, for further details, for example, body composition) were recruited but were generally healthy. All study participants have given their written consent to the use of their data. Furthermore, elite athletes, individuals who participated vigorously in competitive sports (for the past 2 years), and anyone with a history of chronic illness or medication use have been excluded from participation. The participants received a 12-week training intervention that included both endurance and strength training, as previously published by Krammer et al. 2022 [55]. During their training sessions, they were supervised by the gym trainers and our staff and had to keep a training log. The participants were contacted weekly by phone and asked about their progress or motivation when needed. Before (T0) and after (24–48 hours after the last training session, T1) the intervention, capillary blood samples (DBS) were collected, the body composition was measured (using bioelectrical impedance analysis (BIA) to measure basal metabolic rate, phase angle, LBM, BFM, etc.), and a questionnaire regarding diet and lifestyle was queried. Sixty-one participants (20 men and 41 women) completed the intervention and in addition, capillary blood samples were collected from nine participants 10 months after the intervention (T2, Table S2) to see whether the expressions returned to baseline (Figure 1B).

To exclude seasonal or diet-related changes in expression patterns, 20 participants were examined as a control group, women and men, aged between 23 and 65 years, and with a BMI between 20 and 30 kg/m^2^, without any sports intervention or lifestyle change (Figure 1B and Table S3).

### Bioelectrical impedance analysis (BIA)

Body composition was assessed using a multi-frequency (5, 50, and 100 kHz) and phase-sensitive BIA. The measurement device Nutriguard-MS (Data Input GmbH, Germany) measures resistance R and reactance Xc. According to the manufacturers’ protocol, study participants were instructed not to eat for 4–5 h, not to consume alcoholic beverages for 24 h, and to refrain from strenuous exercise for 12 h before the analysis. The subjects had to lie down and rest for 5 minutes before measurement. Extremities should have the temperature of regular skin circulation. The participant’s legs should lie apart at approximately 45°, arm should be spread at approximately 30°. They should not touch the rest of the body. One pair of adhesive gel electrodes were placed on the hand and the other pair on the foot. The BIA analysis shows excellent reliability with repeat measurements [19].

### Sample collection, RNA- and DNA extraction

The capillary blood was collected on *Whatman® protein saver cards* (Sigma-Aldrich, Austria) using the *safety Lancet Extra 18 G* (Sarstedt, Germany) from the fingertip of the non-dominant hand and dried overnight. Two points with a diameter of 10 mm were punched out of each DBS. Then, the RNA was extracted using the *miRNeasy Micro Kit* (Qiagen, Germany) and the DNA using *the QIAamp® DNA Mini Kit* (Qiagen, Germany). Samples were stored at −20 °C. Twelve RNA samples from the pilot study were sent to Qiagen in Germany for further analysis (*miRCURY LNA miRNA PCR Human Panel I*, Cat. No./ID, 339,322 and YAHS-301).

### SNP genotyping

We used the *TaqMan^TM^ Genotyping Assays* from Thermofisher, Netherlands for the analysis of the SNPs (*ACTN3* (rs1815739), *ACE* (rs4341), and *FTO* (rs1121980)) under the default settings on *QuantStudio^TM^ 3*. The SNPs were then evaluated using the *TaqMan Genotyper Software v1.6*.

### Bisulfite conversion and high-resolution melting (HRM) analysis

The DNA samples were prepared with the *EpiTect Bisulfite Kit* (Qiagen, Germany) for the qPCR and HRM analysis using Rotor Gene® Q. For the *Line-1* methylation analysis, we used the primers from Marques-Rocha et al., 2016 [20]. The methylation percentage was then calculated using the AUC method.

### cDNA-Synthesis and real-time PCR (qPCR)

We used the *TaqMan^TM^ Advanced microRNA cDNA Synthesis Kit* and *TaqMan^TM^ Advanced microRNA assays* under the default settings on *QuantStudio^TM^ 3* from Thermofisher, Netherlands. The cDNA samples were stored at −20 °C. In the pilot study, miR-21-5p and miR-155-3p were analyzed in addition to the Qiagen panel. Based on this study, eight miRNAs (miR-19b-3p, −20a-5p, −22-5p, −30e-3p, −101-3p, −146a-5p, −378a-3p and −505-3p) were then included and analyzed in the sports intervention study 2019 – 2020. Furthermore, two reference genes (miR-24-3p and −93-5p) were examined for the evaluation of the expression patterns (using the ∆∆Ct method) of the eight selected miRNAs. The reference genes were selected based on the pilot study and through the literature.

### Statistical analysis

The statistical evaluation was carried out using *IBM SPSS statistics 20* and *GraphPad Prism 6*. Paired t-tests and ANCOVA (adjusted to baseline) were used to compare the different time points. Correlations between miRNA expressions and genotypes were tested using one-way ANOVA (Bonferroni and Scheffé). Correlations between miRNA expressions, *Line-1* methylation, and body composition were tested using Pearson correlation and linear regression, while those between nutritional habits and miRNA expressions were tested using Spearman’s Rho correlation and Kendall’s Tau. For all tests, a p-value equal to or less than 0.05 was assumed to be significant.

## Results

### Pilot study 2018

In total, 12 out of the 14 samples were sent to Qiagen for miRNA analysis (Figure 1A, red box “miRCURY LNA miRNA PCR Human Panel I, Qiagen”) revealing eight significant altered miRNAs (miR-494-3p, −369-5p, −122-5p, −135a-5p, −146a-5p, −30e-3p, −22-5p, and −20a-5p; p < 0.05) after the intervention. Four of these miRNAs (miR-146a-5p, −30e-3p, −22-5p, and −20a-5p) were altered uniformly in all 12 samples. These miRNAs and four others (miR-19b-3p, −101-3p, −505-3p, and −378a-3p, p < 0.07), which also changed in all samples, but not significantly, were then analyzed in the sports intervention study. Furthermore, *Line-1* methylation (n, 14, p, 0.003, Figure 1A, red box “In-house Analysis”) increased significantly, which was also analyzed in the following study.

### Intervention study 2019 – 2020

The selected miRNAs and DNA methylation sites were analyzed in the control group and intervention group before (T0) and after (T1) 12 weeks. Since training adjustments require several weeks, even up to 3 months for muscle building [21], and to provide a “buffer”, for example, if a participant has a cold for a week, which could affect or interrupt their training, we decided to extend the study duration of the following study by 2 weeks. To assess the persistent consequence of the intervention, analysis was also done 10 months after the intervention (T2) in the follow-up group. SNP genotyping was carried out in the intervention and control group.

#### Results of the selected miRNAs and *Line-1* methylation

##### Intervention group

Five of the selected miRNAs showed an altered expression level after the sports intervention. Three miRNAs (miR-20a-5p, p, 0.017, 0.95-fold, miR-22-5p, p, 0.012, 0.95-fold and miR-505-3p, p, 0.006, 0.95-fold) were significantly downregulated and two (miR-30e-3p, p, 0.075, 0.97-fold and miR-146a-5p, p, 0.066, 0.97-fold) showed a downregulation trend. Adjusting to baseline supports the results for miR-20a-5p, −22-5p, −30e-3p, and −505-3p. Additional adjustment for age did not change the effects of sports on these miRNAs and we could exclude age as a confounder (Table 1). However, to quantify the effect of the intervention and because a single molecular biomarker alone is not as meaningful as a combined analysis, we evaluated a “fitness score” that depends on the strength/p-values of the measured miRNAs in the intervention and control group, and determined using the following formula,

$$\Omega =\overset{8}{\underset{i=1}{\sum }}\left(\Delta Ct_{i}*ln\frac{p_{i}^{C}}{p_{i}^{I}}\right)$$

**Table 1.** Results of the analyzed markers of the participants of the sports intervention group. Results were given in mean ± SD.

	Total (n, 61)			Male (n, 20)		Female (n, 41)	
	Fold Change ± SD	Paired t-test	ANCOVA	Fold Change ± SD	Paired t-test	Fold Change ± SD	Paired t-test
**miR-19b-3p**	1.212 ± 0.764	0.463	0.192	1.119 ± 0.619	0.641	1.221 ± 0,832	0.576
**miR-20a-5p**	0.948 ± 0.291	0.017*	0.002*	0.917 ± 0.265	0.083	0.963 ± 0.305	0.095
**miR-22-5p**	0.945 ± 0.315	0.012*	0.010*	0.891 ± 0.311	0.034*	0.971 ± 0.318	0.123
**miR-30e-3p**	0.973 ± 0.290	0.075	0.059	1.010 ± 0.330	0.568	0.954 ± 0.271	0.072
**miR-101-3p**	1.424 ± 1.241	0.256	0.265	1.744 ± 1.537	0.280	1.268 ± 1.054	0.621
**miR-146a-5p**	0.967 ± 0.297	0.066	0.258	0.905 ± 0.290	0.114	0.998 ± 0.298	0.345
**miR-378a-3p**	0.985 ± 0.248	0.172	0.397	0.974 ± 0.213	0.315	0.991 ± 0.266	0.313
**miR-505-3p**	0.948 ± 0.177	0.006*	0.016*	0.923 ± 0.127	0.011*	0.961 ± 0.197	0.075
***Line-1*** methylation	−0.306 ± 4.166	0.568	0.851	−0.448 ± 3.818	0.606	−0.238 ± 4.369	0.729

**Abbreviations**: SD, standard deviation, *Shows significant p-values.

**Figure 2.**
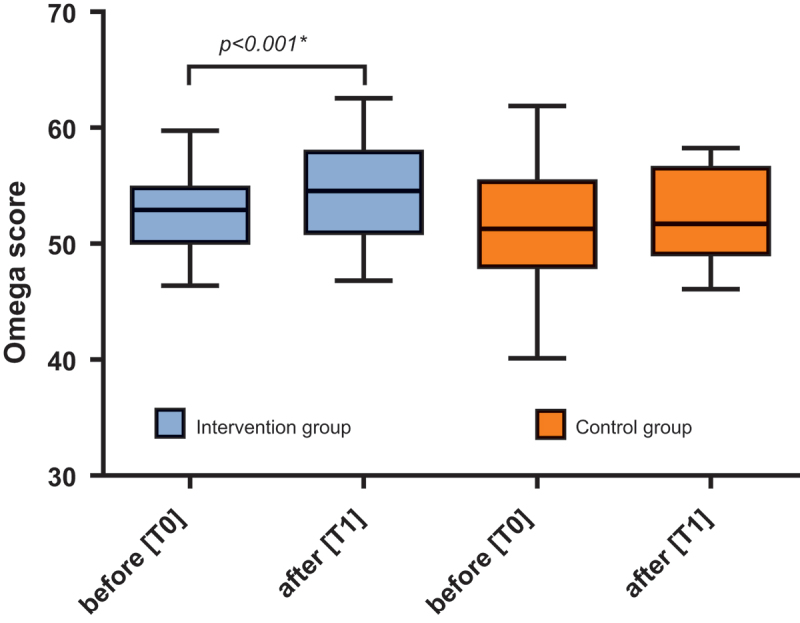
Fitness/Ω score of the intervention group (n, 61) and the control group (n, 20) before [T0] and after [T1]. *Shows significant p-values (paired t-test).

[22] where *∆Ct_i_* is the respective miRNA, $p_{i}^{C}$ is the p-value of the control group and $p_{i}^{I}$ is the p-value of the intervention group. Where a high score indicates a very small p-value in the intervention group and means better fitness, and as can be seen in Figure 2, the “fitness score” increased significantly in the entire intervention group (p, 0.000, effect size, 0.521), in men (p, 0.005) and women (p, 0.009), while it did not change in the control group (p, 0.740). Furthermore, the methylation of *Line-1* showed no change after the 12-week intervention.

##### Control group

In the control group, only one miRNA had a changed expression after 12 weeks. The miR-20a-5p showed a significant upregulation (p, 0.020, 1.48-fold), whereas in the intervention group a significant downregulation was observed. When the intervention and the control group are compared, they showed a significantly different expression of miR-20a-5p (p, 0.042) before the intervention (T0), and significantly different expressions of miR-22-5p (p, 0.000), −30e-3p (p, 0.015), and −146a-5p (p, 0.047) after 12 weeks (T1). If considering the fold changes of the two groups, they differ significantly in miR-20a-5p (p, 0.012) and miR-22-5p (p, 0.046).

##### Follow-up group

In the follow-up group, we observed an upregulated miR-378a-3p 10 months after the sports intervention (T2), compared to immediately after the intervention (T1) (p, 0.050). When compared before intervention (T0) and 10 months after the intervention (T2), there were no differences in the expression levels of these miRNAs.

#### Results of the SNP genotyping

Table 2 shows the distribution of the selected single nucleotide polymorphisms (SNPs) in the respective genes of our study participants in the intervention (n, 61) and control group (n, 20).

**Table 2.** Distribution of the SNPs in the *FTO* (rs1121980), *ACE* (rs4341), and *ACTN3* (rs1815739) gene. Genotyping values are given in total numbers and as a percentage.

Gene	SNP ID			
*FTO*	rs1121980	Homozygotes (GG)	Homozygotes (AA)	Heterozygotes (AG)
Intervention	Total, % (n)	29.5 % (18)	24.6 % (15)	45.9 % (28)
	Male, % (n)	20.0 % (4)	30.0 % (6)	50.0 % (10)
	Female, % (n)	34.1 % (14)	22.0 % (9)	43.9 % (18)
Control	Total, % (n)	30.0 % (6)	25.0 % (5)	45.0 % (9)
	Male, % (n)	57.1 % (4)	0 % (0)	42.9 % (3)
	Female, % (n)	15.4 % (2)	38.5 % (5)	46.2 % (6)
***ACE***	**rs4341**	**Homozygotes (CC)**	**Homozygotes (GG)**	**Heterozygotes (CG)**
Intervention	Total, % (n)	19.7 % (12)	31.1 % (19)	49.2 % (30)
	Male, % (n)	20.0 % (4)	30.0 % (6)	50.0 % (10)
	Female, % (n)	19.5 % (8)	31.7 % (13)	48.8 % (20)
Control	Total, % (n)	5.0 % (1)	25.0 % (5)	70.0 % (14)
	Male, % (n)	14.3 % (1)	14.3 % (1)	71.4 % (5)
	Female, % (n)	0 % (0)	30.8 % (4)	69.2 % (9)
***ACTN3***	**rs1815739**	**Homozygotes (CC)**	**Homozygotes (TT)**	**Heterozygotes (TC)**
Intervention	Total, % (n)	32.8 % (20)	18.0 % (11)	49.2 % (30)
	Male, % (n)	20.0 % (4)	30.0 % (6)	50.0 % (10)
	Female, % (n)	39.0 % (16)	12.2 % (5)	48.8 % (20)
Control	Total, % (n)	35.0 % (7)	30.0 % (6)	35.0 % (7)
	Male, % (n)	57.1 % (4)	14.3 % (1)	28.6 % (2)
	Female, % (n)	23.1 % (3)	38.5% (5)	38.5 % (5)

**Abbreviations:** FTO, Fat mass and obesity-associated protein, ACE, Angiotensin-converting enzyme, ACTN3, alpha-Actinin 3.

##### Differences between genotypes and selected miRNAs

**Figure 3.**
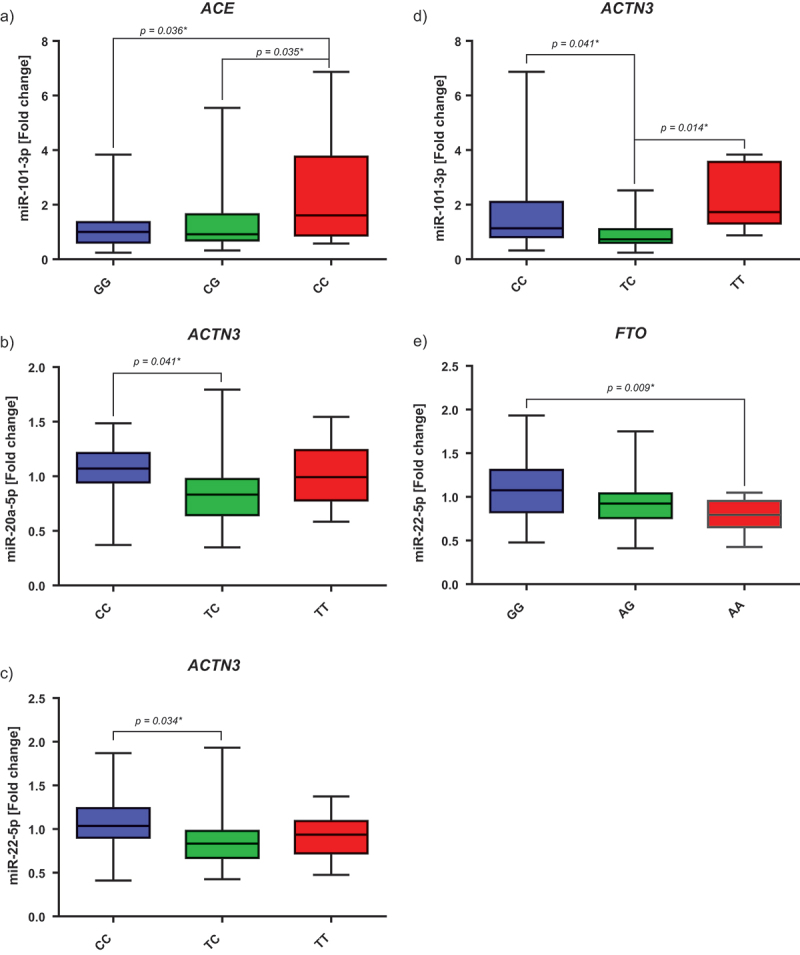
Fold changes of the selected miRNAs by genotype. **A)** Fold change of miR-101-3p by *ACE* genotype (GG strength, CC endurance). **B)** Fold change of miR-20a-5p by *ACTN3* genotype (CC strength, TT endurance). **C)** Fold change of miR-22-5p by *ACTN3* genotype (CC strength, TT endurance). **D)** Fold change of miR-101-3p by *ACTN3* genotype (CC strength, TT endurance). **E)** Fold change of miR-22-5p by *FTO* genotype (AA risk variant). The p-values given here are the results of the post hoc tests (Scheffé). * Shows significant p-values.

Polymorphisms in the *ACE* gene seem to influence the expression of miR-101-3p (p, 0.019). Participants with a CC genotype had a significantly higher expression after the intervention compared to participants with a CG or GG phenotype, with no differences between CG and GG (Figure 3A). Furthermore, polymorphisms in the *ACTN3* gene appear to have an influence on the expression of miR-20a-5p (p, 0.027), miR-22-5p (p, 0.033), and miR-101-3p (p, 0.004), see Figure 3B-D. Participants with a CC genotype had a significantly higher expression of these miRNAs after 12 weeks than those with a TC genotype. Whereas the participants with a TT genotype showed a higher expression of miR-101-3p than TC genotypes. Furthermore, the polymorphism in the *FTO* gene also seems to influence the expression of miR-22-5p (p, 0.008). However, only the GG genotype differed from the AA genotype. Participants with a GG genotype showed a higher expression after the intervention than those with an AA genotype (Figure 3E). In the control group, we could not detect any significant correlations between the different SNP genotypes and the individual miRNAs (p > 0.05).

#### Results of the body composition measurement (BIA)

The Intervention resulted in significant changes in the anthropometric data. There was an increase in basal metabolic rate (p, 0.000), phase angle (p, 0.004), LBM (p, 0.000), BCM (p, 0.007), as well as ICW (p, 0.007), and ECW (p, 0.015), and a decrease in BFM (p, 0.000), see Table 3.

**Table 3.** Body composition changes through the 12-week sports intervention. Anthropometric measurements were given in mean ± SD.

	Total (n, 61)		Male (n, 20)		Female (n, 41)	
	Change ± SD	p-Value	Change ± SD	p-Value	Change ± SD	p-Value
Basal metabolic rate [kcal]	23.10 ± 41.74	0.000*	20.45 ± 25.67	0.002*	24.39 ± 47.91	0.002*
Phase angel [°]	0.16 ± 0.42	0.004*	0.07 ± 0.25	0.232	0.21 ± 0.48	0.009*
LBM [kg]	0.68 ± 1.39	0.000*	0.84 ± 1.32	0.010*	0.60 ± 1.43	0.010*
BFM [kg]	−1.14 ± 1.87	0.000*	−1.33 ± 2.19	0.014*	−1.04 ± 1.72	0.000*
BCM [kg]	0.77 ± 2.16	0.007*	0.64 ± 3.17	0.378	0.83 ± 1.48	0.001*
ICW [l]	0.31 ± 0.46	0.000*	0.33 ± 0.43	0.003*	0.30 ± 0.48	0.000*
ECW [l]	0.20 ± 0.63	0.015*	0.29 ± 0.64	**0.056**	0.16 ± 0.63	0.112

**Abbreviations**: LBM, lean body mass, BFM, body fat mass, BCM, body cell mass, ICW, intracellular water, ECW, extracellular water. Fold Change, mean difference between the 2 timepoints, SD, standard deviation. *Shows significant p-values (paired t-test).

##### Epigenetic and genetic markers and anthropometric data

**Figure 4.**
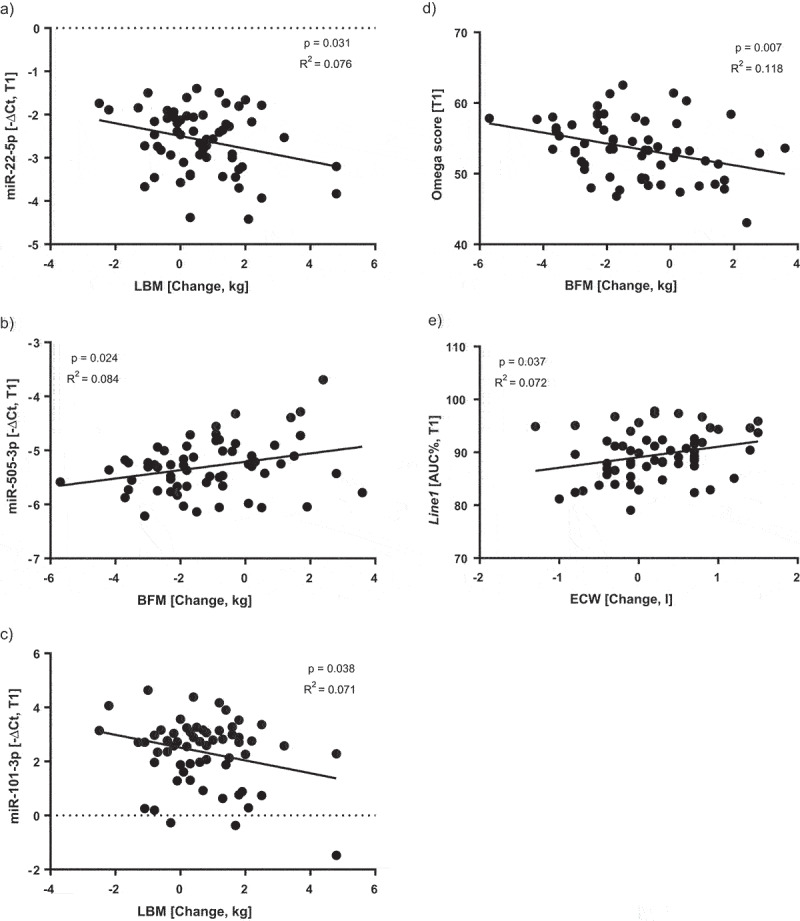
Significant correlations between the change of the anthropometric data and the expression of selected miRNAs, the fitness/Ω score, and *Line-1* methylation after the Intervention [T1]. **A)** Correlation between the change of LBM and the expression of miR-22-5p at T1. **B)** Correlation between the change of BFM and the expression of miR-505-3p at T1. **C)** Correlation between the change of LBM and the expression of miR-101-3p at T1. **D)** Correlation between the change of BFM and the fitness/Ω score at T1. **E)** Correlation between the change of ECW and the *Line-1* methylation at T1. Statistical significance was defined as a p-value below 0.05.

We were also able to determine significant correlations between miRNAs, *Line-1* methylation, and anthropometric data. Between miR-22-5p (linear regression, −0.277, p, 0.031), miR-101-3p, and LBM (linear regression, −0.267, p, 0.038). Among miR-505-3p and BFM (linear regression, 0.290, p, 0.024), between *Line-1* methylation and ECW (linear regression, −0.267, p, 0.037), and among the ”fitness score” and BFM (linear regression, −0.343, p, 0.007), see Figure 4. For example, the participants who gained more LBM had a lower expression of miR-22-5p (Figure 4A) and miR-101-3p (Figure 4C), and participants who lost more BFM had a lower expression of miR-505-3p and a higher ”fitness score” (Figure 4 B and D).

Furthermore, we could observe correlations between *ACTN3* gene, LBM (p, 0.039), ICW (p, 0.040), and BMI (p, 0.072). The participants with a TC genotype showed a higher gain in LBM and ICW compared to participants with a TT genotype.

#### Results of epigenetic markers, nutrition, and lifestyle

In the intervention group, we were able to determine a significant correlation between the intake of B vitamins (B vitamin complex, cobalamin, and folic acid), miR-19b-3p (p, 0.033), and miR-101-3p (p, 0.047). Participants who supplemented with B vitamins had a higher expression of these miRNAs compared to participants who did not supplement. A similar correlation could be established between the intake of magnesium and miR-19b-3p (p, 0.052). Furthermore, we were able to observe an association between miR-378a-3p and iron supplementation (p, 0.017). The participants who supplemented iron had a significantly higher expression of miR-378a-3p. In addition, the participants who had a higher fluid intake (2–3 liters a day) showed an upregulation of miR-378a-3p (p, 0.003). Furthermore, the participants who reported consuming whole grain products daily or several times a day showed a higher expression level of miR-101-3p (p, 0.002) than those who consumed less. Whereas miR-146a-5p correlated with stress (p, 0.029). The participants who reported having an increased stress level had a significantly lower expression of miR-146a-5p.

## Discussion

The main aim of this study was to measure training effects via miRNAs from dried blood spots (DBS) and to determine how miRNAs are regulated by medium-term exercise (strength and endurance training), according to Ashton et al. 2020 [23], in sedentary, healthy individuals. But also, what role genetic predisposition or nutrition plays, and the possible interactions with the selected miRNAs, as well as the development of a biomarker algorithm that provides information about the performance, health, and recovery of an athlete.

As far as we know, we are among the first to describe a so-called miRNA-based “fitness score” biomarker in capillary blood, which can describe a person’s current fitness level regardless of age, sex, and genetic predisposition. And even if some of the miRNAs analyzed in this study have already been described in combination with exercise, and could be used as biomarkers, such as miR-20a and −146a for cardiorespiratory fitness and peak exercise capacity [24], a combination of several miRNAs promises a more precise assessment of health and training levels. Also, with the current commonly used biomarkers, it was found that a single maker alone is too meaningless and that a combined analysis of several parameters provides much more accurate information and thus helps athletes or coaches to optimize training and prevent overtraining [16]. Furthermore, using a miRNA-based biomarker algorithm would have the further advantage that the miRNA expression patterns change much earlier than markers from proteins and/or electrolytes [17]. For this purpose, it should also be considered which information our miRNAs provide, which sport-relevant physiological functions they may reflect or have and where they come from.

It is known that exercise training leads to an increased rate of red blood cell (RBC) breakdown due to oxidative stress. However, as a result, exercise training stimulates erythropoiesis and increases RBC mass, the number of leukocytes, hematocrit values, and hemoglobin concentrations, as well as the plasma volume. And, an increased rate of RBC turnover can be beneficial as young cells are more efficient at transporting oxygen [25–27]. Moreover, it was found that RBCs are the leading cause of miRNA expression in whole blood and that the amount of erythrocyte-derived miRNAs reflects the majority of miRNAs expressed in the whole blood. It is described that these RBC-derived miRNAs show high-expression patterns, and some are delivered to recipient cells via extracellular erythrocyte vesicles and are thus involved in the regulation of gene expression. However, the amount of the endogenous controls (miR-24-3p and −93-5p) in our DBS samples did not change during the intervention, and we could not find any indications in the literature that they are involved in the regulation of erythropoiesis in healthy humans. Moreover, there are other important cell types in the whole blood, such as leukocytes and platelets, which contain functional miRNAs [28].

As already indicated, exercise also affects leukocytosis and increases the number of leukocytes and neutrophils. It is believed that these changes in the hemodynamics of blood cells take place in the circulatory system and may not weaken the immune system but instead strengthen it due to the increased number of immune cells. Although exercise can resemble acute inflammation by recruiting leukocytes, this could be beneficial as most cells are recruited into the bloodstream rather than into inflamed tissues, as in infection [29]. The analyzed miRNAs may come from leukocytes or neutrophils, since exercise can also alter the miRNA expression patterns in neutrophils in the circulating blood and thus possibly influence the function of neutrophils [30]. Furthermore, it was shown that miR-20a and −22 were downregulated in neutrophils after endurance training, while miR-505 was upregulated, and miR-30e in natural killer cells and monocytes. Some of the neutrophil-derived miRNAs, including miR-20a, are also known to regulate genes involved in immune processes and apoptosis, such as Jak-STAT signaling pathways [15,30–32]. Also, miR-146a plays an essential role in the inflammatory signaling in several cell types, and it was concluded that changes in the circulating miR-146a could reflect the overall inflammatory state that is seen in athletes who engage in prolonged aerobic exercise [33]. Moreover, this miRNA also correlates with the inflammation marker high-sensitivity C-reactive protein [34]. In summary, this suggests that some of our miRNAs play a role in the immune response that is triggered by exercise.

However, we were able to determine differences in the expression of miR-20a-5p, −22-5p, and −101-3p depending on the *α-actinin-3* gene (*ACTN3)*, often also referred to as the “speed gene”. Subjects with a CC (or RR) genotype had a higher expression of miR-20a-5p and −22-5p after 12 weeks than those with a TC (or XR) genotype, while participants with a TT (or XX) genotype had a higher expression of miR-101-3p than those with a TC or CC genotype. The protein ACTN3 is only expressed in the fast type II fibers and is related to the generation of fast and powerful muscle movements. It also appears to play a role in adaption and recovery after exercise and in the risk of injury. In contrast to individuals with TC or CC, those with homozygous TT cannot express ACTN3, which can lead to impaired muscle performance and make them predisposed to muscle damage and ligament injuries during exercise [7]. In animal studies, but also in some human studies, especially in women, this phenotype has been also associated with increased endurance capacity [35–37]. Our results indicate that there may be an association between the expression of miR-20a-5p, −22-5p, and −101-3p, the genotype of *ACTN3*, and the ability to perform fast and explosive movements and/or the injury risk. Additionally, we were able to observe a correlation between miR-22-5p, −101-3p and the increase in lean body mass, which further confirms a possible connection to jumping power or explosive movements. It is also discussed that these miRNAs are involved in cardiac hypertrophy triggered by endurance exercise, and we suspected that they may also have pro-/anti-hypertrophic effects in other tissues [38]. Furthermore, for participants with the *FTO* risk allele (AA), the expression of miR-22-5p was downregulated. One known target of miR-22 is the *histone deacetylase 4* (*HDAC4*) [39], supporting the theory that metabolic phenotypes are probably mediated by epigenetic mechanisms due to the different *FTO* mutations [40]. These results highlight the importance of personalized analysis and interventions, as the epigenetic phenotype varies significantly depending on the genotype.

Anyway, it is known that exercise-induced muscle injuries and damage often occur after unusual or intense training, especially if the training involves many eccentric contractions [41,42]. In response to damage, activated satellite cells begin to multiply and undergo myogenic differentiation as part of the muscle regeneration process. The proliferation and differentiation of satellite cells are associated with changes in miRNA expression, which leads to an altered expression of target genes that are important for muscle regeneration. These muscle-specific miRNAs also include miR-378a-3p, which has been identified as a promising marker for acute muscle damage [43,44]. Furthermore, miR-378a-3p is a positive regulator of osteogenesis and is therefore involved in bone formation and resorption, making miR-378a-3p a marker of bone health and injury/bone fracture risk [45,46]. Other miRNAs are also involved in the regulation of bone remodeling and regeneration, such as miR-146a-5p, which, in contrast to miR-378a-3p, suppresses osteoblastogenesis [47], and miR-30e, which inhibits osteoblast differentiation, whereby its expression level maintains the balance between osteoblast and adipocyte differentiation [48]. Bone marrow adipocytes influence bone remodeling and play a role in bone loss disorders [49]. However, exercise can suppress bone marrow adipocyte expansion [50], maybe through miR-30e.

Besides these findings, in our study, we were also able to observe a correlation between the self-reported supplementation of B vitamins, which include thiamine, riboflavin, niacin, pantothenic acid, pyridoxine, biotin, and folic acid or folate, and miR-19b-3p expression. An intake of B vitamins leads to an upregulation of miR-19b-3p. Furthermore, we could also observe that the participants who supplemented magnesium had a higher expression of miR-19b-3p, and those who consumed iron supplements had a higher expression of miR-378a-3p. Very little evidence of such correlations can be found in the literature. But it is known that micronutrients are essential for maintaining the health of physically active people and that especially B vitamins are essential for energy production, synthesis of new cells, and repair of damaged cells. Whereas others are necessary for maintaining immune function, bone health, and protecting against oxidative damage, but also for building and repairing muscle tissue. Both biochemical adaptions to exercise and the metabolic pathways for energy production can increase the need for certain micronutrients during regular physical activity, and athletes with a poor B vitamin status may have a decreased ability to exercise at a high-intensity level [51–53]. Furthermore, it is important particularly for athletes to maintain an optimal micronutrient status and, if necessary, to recognize a deficiency at an early stage before physical signs appear. Additionally, the miR-378a-3p expression correlated with the amount of average fluid intake. During exercise, especially at high temperatures, sweating and inadequate hydration can lead to dehydration [16]. And there are indications that moderate hypohydration, even in short phases, can lead to disturbance of cognitive functions, a reduction in the ability to concentrate as well as headaches or fatigue, and thus impair the performance of an athlete [54]. In summary, the correlations between micronutrients, fluid intake, and miRNAs observed in our study indicate that some miRNAs are suitable for reflecting the current micronutrient and hydration status of an athlete, as predictive biomarkers.

The results of our study reported here should be considered in light of some limitations. First, the effects of concurrent strength and endurance exercise were examined since the general population tends to engage in both types of exercise. However, future studies should also consider the individual training methods alone, with each person completing three intervention cycles, strength, endurance, and concurrent. Second, the study focused on a wide age range. The main goal compared to most previous studies was to develop a minimally invasive method with biomarkers that are suitable for the general population, not just for young people or elite athletes. Although we could exclude age as a confounder, this should be considered in future studies. Nevertheless, our minimally invasive method offers the advantage that anyone, amateurs, and elite athletes, can easily take samples anywhere and at any time to monitor their molecular response to exercise.

## Conclusions

**Figure 5.**
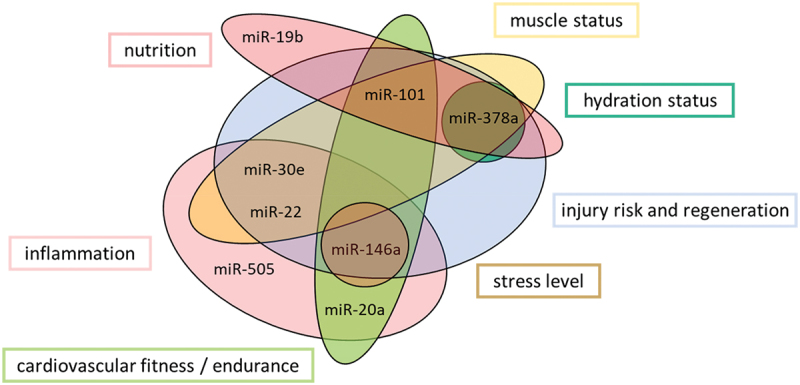
The properties of the measured miRNAs and their importance and classification as sports-relevant biomarkers.

In conclusion, our “fitness score” not only reflects the effect of the intervention, moreover, but the individual miRNAs also provide information to assess the health, performance, and recovery of an athlete/person. Based on our findings, correlations between miRNAs, SNPs, body composition, diet, and the literature, we have divided the miRNAs, according to the work by Lee et al., 2017 [16], into the following subgroups (Figure 5), nutrition, inflammation, cardiovascular fitness, injury risk, regeneration, muscle- and hydration status, as well as stress level. As a result, our biomarker enables easier training tracking, as samples can be taken anytime and anywhere (DBS), and athletes and coaches can make possible adjustments at an early stage to optimize performance or prevent deficits.

## Supplementary Material

Supplemental Material

Supplemental Material

Supplemental Material

## Abbreviations

ACE, Angiotensin-converting enzyme, ACTN3, Alpha-actin-3, AUC, Area under the curve, BCM, Body cell mass, BFM, Body fat mass, BIA, Bioelectrical impedance analysis, BMI, Body mass index, DBS, Dried blood spot, ECW, Extracellular water, FFQ, Food frequency questionnaire, FTO, Fat mass and obesity-associated gene, HRM, High-resolution melting, ICW, Intracellular water, LBM, Lean body mass, miRNA, microRNA, MREs, miRNA response elements, qPCR, Real-time polymerase chain reaction, RBC, Red blood cell, SNP, Single nucleotide polymorphism

## References

[1] Mikkelsen K, Stojanovska L, Polenakovic M, et al. Exercise and mental health. Maturitas. 2017;106:48–56. 29150166 10.1016/j.maturitas.2017.09.003

[2] Radak Z, Torma F, Berkes I, et al. Exercise effects on physiological function during aging. Free Radic Biol Med. 2019;132:33–41. 30389495 10.1016/j.freeradbiomed.2018.10.444

[3] Distefano G, Goodpaster BH. Effects of exercise and aging on skeletal muscle. Cold Spring Harb Perspect Med. 2018;8(3):a029785. 28432116 10.1101/cshperspect.a029785 PMC5830901

[4] Ling C, Rönn T. Epigenetic adaptation to regular exercise in humans. Drug Discov Today. 2014;19(7):1015–1018. 24632002 10.1016/j.drudis.2014.03.006

[5] Kelly SA, Pomp D. Genetic determinants of voluntary exercise. Trends Genet. 2013;29(6):348–357. 23351966 10.1016/j.tig.2012.12.007 PMC3665695

[6] Herring MP, Sailors MH, Bray MS. Genetic factors in exercise adoption, adherence and obesity. Obes Rev. 2014;15(1):29–39. 24034448 10.1111/obr.12089

[7] Del Coso J, Hiam D, Houweling P, et al. More than a ‘speed gene’, ACTN3 R577X genotype, trainability, muscle damage, and the risk for injuries. Eur J Appl Physiol. 2019;119(1):49–60. 30327870 10.1007/s00421-018-4010-0

[8] Heffernan SM, Stebbings GK, Kilduff LP, et al. Fat mass and obesity associated (FTO) gene influences skeletal muscle phenotypes in non-resistance trained males and elite rugby playing position. BMC Genet. 2017;18(1):1–9. 28103813 10.1186/s12863-017-0470-1 PMC5248469

[9] Almén MS, Jacobsson JA, Moschonis G, et al. Genome wide analysis reveals association of a FTO gene variant with epigenetic changes. Genomics. 2012;99(3):132–137. 22234326 10.1016/j.ygeno.2011.12.007

[10] Grazioli E, Dimauro I, Mercatelli N, et al. Physical activity in the prevention of human diseases, role of epigenetic modifications. BMC Genomics. 2017;18(S8). DOI:10.1186/s12864-017-4193-5. PMC5688489 29143608

[11] Voisin S, Eynon N, Yan X, et al. Exercise training and DNA methylation in humans. Acta Physiol. 2015;213(1):39–59. 10.1111/apha.12414 25345837

[12] McGee SL, Hargreaves M. Epigenetics and exercise. Trends Endocrinol Metab. 2019;30(9):636–645. 31279665 10.1016/j.tem.2019.06.002

[13] Kern F, Ludwig N, Backes C, et al. Systematic assessment of blood-borne MicroRNAs highlights molecular profiles of endurance sport and carbohydrate uptake. Cells. 2019;8(9):1045. 10.3390/cells8091045 PMC6770460 31500139

[14] Wang H, Liang Y, Li Y. Non-coding RNAs in exercise. Non-coding RNA Investig. 2017;1:10.

[15] Silva GJJ, Bye A, El Azzouzi H, et al. MicroRNAs as important regulators of exercise adaptation. Prog Cardiovasc Dis. 2017;60(1):130–151. 28666746 10.1016/j.pcad.2017.06.003

[16] Lee EC, Fragala MS, Kavouras SA, et al. Biomarkers in sports and exercise, tracking health, performance, and recovery in athletes. J Strength Cond Res. 2017;31(10):2920. 28737585 10.1519/JSC.0000000000002122 PMC5640004

[17] Moldovan L, Batte KE, Trgovcich J, et al. Methodological challenges in utilizing mi RNA s as circulating biomarkers. J Cell Mol Med. 2014;18(3):371–390. 24533657 10.1111/jcmm.12236 PMC3943687

[18] Simmonds MJ, Baskurt OK, Meiselman HJ, et al. A comparison of capillary and venous blood sampling methods for the use in haemorheology studies. Clin Hemorheol Microcirc. 2011;47(2):111–119. 21339631 10.3233/CH-2010-1372

[19] Von Hurst PR, Walsh DCI, Conlon CA, et al. Validity and reliability of bioelectrical impedance analysis to estimate body fat percentage against air displacement plethysmography and dual-energy X-ray absorptiometry. Nutr Diet. 2016;73(2):197–204.

[20] Marques-Rocha JL, Milagro FI, Mansego ML, et al. LINE-1 methylation is positively associated with healthier lifestyle but inversely related to body fat mass in healthy young individuals. Epigenetics. 2016;11(1):49–60. 26786189 10.1080/15592294.2015.1135286 PMC4846126

[21] Abe T, DeHoyos D V, Pollock ML, et al. Time course for strength and muscle thickness changes following upper and lower body resistance training in men and women. Eur J Appl Physiol Occup Physiol. 2000;81(3):174–180. 10.1007/s004210050027 10638374

[22] Cohen JD, Li L, Wang Y, et al. Detection and localization of surgically resectable cancers with a multi-analyte blood test. Science. 2018;359(6378):926–930. 29348365 10.1126/science.aar3247 PMC6080308

[23] Ashton RE, Tew GA, Aning JJ, et al. Effects of short-term, medium-term and long-term resistance exercise training on cardiometabolic health outcomes in adults, systematic review with meta-analysis. Br. J. Sports Med. 2020;54(6):341–348. 29934430 10.1136/bjsports-2017-098970

[24] Baggish AL, Hale A, Weiner RB, et al. Dynamic regulation of circulating microRNA during acute exhaustive exercise and sustained aerobic exercise training. J Physiol. 2011;589(16):3983–3994. 21690193 10.1113/jphysiol.2011.213363 PMC3179997

[25] Smith JA. Exercise, training and red blood cell turnover. Sport Med. 1995;19(1):9–31. 10.2165/00007256-199519010-00002 7740249

[26] Hu M, Lin W. Effects of exercise training on red blood cell production, Implications for anemia. Acta Haematol. 2012;127(3):156–164. 22301865 10.1159/000335620

[27] Belviranli M, Okudan N, Kabak B. The effects of acute high-intensity interval training on hematological parameters in sedentary subjects. Med. Sci. 2017;5:15. 10.3390/medsci5030015 PMC5635806 29099031

[28] Sun L, Yu Y, Niu B, et al. Red blood cells as potential repositories of MicroRNAs in the circulatory system. Front Genet. 2020;11:1–8. 32582273 10.3389/fgene.2020.00442 PMC7286224

[29] Sand KL. Effects of exercise on leukocytosis and blood hemostasis in 800 healthy young females and males. World J Exp Med. 2013;3(1):11. 24520541 10.5493/wjem.v3.i1.11 PMC3905589

[30] Radom-Aizik S, Zaldivar F, Oliver S, et al. Evidence for microRNA involvement in exercise-associated neutrophil gene expression changes. J Appl Physiol. 2010;109(1):252–261. 20110541 10.1152/japplphysiol.01291.2009 PMC2904199

[31] Radom-Aizik S, Zaldivar F, Haddad F, et al. Impact of brief exercise on peripheral blood NK cell gene and microRNA expression in young adults. J Appl Physiol. 2013;114(5):628–636. 23288554 10.1152/japplphysiol.01341.2012 PMC3615590

[32] Radom-Aizik S, Zaldivar FP, Haddad F, et al. Impact of brief exercise on circulating monocyte gene and microRNA expression, implications for atherosclerotic vascular disease. Brain Behav Immun. 2014;39:121–129. 24423463 10.1016/j.bbi.2014.01.003 PMC4101903

[33] Baggish AL, Park J, Min PK, et al. Rapid upregulation and clearance of distinct circulating microRNAs after prolonged aerobic exercise. J Appl Physiol. 2014;116(5):522–531. 24436293 10.1152/japplphysiol.01141.2013 PMC3949215

[34] Li Y, Yao M, Zhou Q, et al. Dynamic regulation of circulating microRNAs during acute exercise and long-term exercise training in basketball athletes. Front Physiol. 2018;9:1–11. 29662456 10.3389/fphys.2018.00282 PMC5890107

[35] Roth SM, Walsh S, Liu D, et al. The ACTN3 R577X nonsense allele is under-represented in elite-level strength athletes. Eur J Hum Genet. 2008;16(3):391–394. 18043716 10.1038/sj.ejhg.5201964 PMC2668151

[36] Yang N, MacArthur DG, Gulbin JP, et al. ACTN3 genotype is associated with human elite athletic performance. Am J Hum Genet. 2003;73(3):627–631. 12879365 10.1086/377590 PMC1180686

[37] Yang R, Shen X, Wang Y, et al. ACTN3 R577X gene variant is associated with muscle-related phenotypes in elite Chinese sprint/power athletes. J Strength Cond Res. 2017;31(4):1107–1115. 27442335 10.1519/JSC.0000000000001558

[38] Soplinska A, Zareba L, Wicik Z, et al. MicroRNAs as biomarkers of systemic changes in response to endurance exercise⇔a comprehensive review. Diagnostics. 2020;10(10):1–17. 10.3390/diagnostics10100813 PMC7602033 33066215

[39] Lu W, You R, Yuan X, et al. The microRNA miR-22 inhibits the histone deacetylase HDAC4 to promote TH17 cell-dependent emphysema. Nat Immunol. 2015;16(11):1185–1194. 26437241 10.1038/ni.3292 PMC4597310

[40] Franzago M, Fraticelli F, Marchioni M, et al. Fat mass and obesity-associated (FTO) gene epigenetic modifications in gestational diabetes, new insights and possible pathophysiological connections. Acta Diabetol. 2021;58(8):997–1007. 33743080 10.1007/s00592-020-01668-5 PMC8272710

[41] Clarkson PM, Hubal MJ. Exercise-induced muscle damage in humans. Int. J. Sports Med. 2002;15:132–135. 10.1097/00002060-200211001-00007 12409811

[42] Siracusa J, Koulmann N, Sourdrille A, et al. Phenotype-specific response of circulating miRNAs provides new biomarkers of slow or fast muscle damage. Front Physiol. 2018;9:1–9. 29922177 10.3389/fphys.2018.00684 PMC5996145

[43] Kirby TJ, McCarthy JJ. MicroRNAs in skeletal muscle biology and exercise adaptation. Free Radic Biol Med. 2013;64:95–105. 23872025 10.1016/j.freeradbiomed.2013.07.004 PMC4867469

[44] Siracusa J, Koulmann N, Bourdon S, et al. Circulating miRNAs as Biomarkers of Acute Muscle Damage in Rats. Am J Pathol. 2016;186:1313–1327. 26952641 10.1016/j.ajpath.2016.01.007

[45] Kang M, Huang CC, Lu Y, et al. Bone regeneration is mediated by macrophage extracellular vesicles. Bone. 2020;141:115627. 32891867 10.1016/j.bone.2020.115627 PMC8107826

[46] Amir LR, Everts V, Bronckers ALJJ. Bone regeneration during distraction osteogenesis. Odontology. 2009;97(2):63–75. 19639448 10.1007/s10266-009-0101-z

[47] Chang CC, Venø MT, Chen L, et al. Global MicroRNA profiling in human bone marrow skeletal—stromal or mesenchymal–stem cells identified candidates for bone regeneration. Mol Ther. 2018;26(2):593–605. 29331291 10.1016/j.ymthe.2017.11.018 PMC5835027

[48] Suttamanatwong S. MicroRNAs in bone development and their diagnostic and therapeutic potentials in osteoporosis. Connect. Tissue Res. 2017;58(1):90–102 26963177 10.3109/03008207.2016.1139580

[49] Muruganandan S, Govindarajan R, Sinal CJ. Bone marrow adipose tissue and skeletal health. Curr. Osteoporos. Rep. 2018;16(4):434–442 29855795 10.1007/s11914-018-0451-y PMC6239196

[50] Styner M, Pagnotti GM, McGrath C, et al. Exercise decreases marrow adipose tissue through ß-oxidation in obese running mice. J Bone Miner Res. 2017;32(8):1692–1702. 28436105 10.1002/jbmr.3159 PMC5550355

[51] Woolf K, Manore MM. B-vitamins and exercise, does exercise alter requirements? Int. J. Sport Nutr. Exerc. Metab. 2006;16(5):453–484 17240780 10.1123/ijsnem.16.5.453

[52] Laires MJ, Monteiro C. Exercise, magnesium and immune function. Magnes Res. 2008;21(2):92–96. 18705536

[53] Beard J, Tobin B. Iron status and exercise. Am J Clin Nutr. 2000;72(2):594–597. 10.1093/ajcn/72.2.594S 10919965

[54] Maughan RJ. Impact of mild dehydration on wellness and on exercise performance. Eur J Clin Nutr. 2003;57(S2):S19–S23. 14681709 10.1038/sj.ejcn.1601897

[55] Krammer UDB, Sommer A, Tschida S, Mayer A, Lilja SV, Switzeny OJ, Hippe B, Rust P and Haslberger A G. (2022). PGC-1α Methylation, miR-23a, and miR-30e Expression as Biomarkers for Exercise- and Diet-Induced Mitochondrial Biogenesis in Capillary Blood from Healthy Individuals: A Single-Arm Intervention. Sports, 10(5), 73 10.3390/sports10050073 35622482 PMC9143572

