# Supplementary material for: MiRNA-based “fitness score” to assess the individual response to diet, metabolism, and exercise
Source: J Int Soc Sports Nutr. 2022 Aug 2;19(1):455–73. doi: 10.1080/15502783.2022.2106148 (PMC9351578; doi:10.1080/15502783.2022.2106148)
Supplement: Supplemental Material [file RSSN_A_2106148_SM7980.pdf]

	Male	Female	Total
**n**	7	13	20
**Age [years]**	36.29 ± 13.61	39.31 ± 10.00	38.25 ± 11.13
**Age range [years]**	25 - 65	23 - 60	23 - 65
**BMI [T0, kg/m^2]**	25.16 ± 2.16	24.87 ± 2.73	24.97 ± 2.49
**BMI [T1, kg/m^2]**	25.26 ± 2.51	24.89 ± 3.12	25.03 ± 2.83
