# Supplementary material for: MiRNA-based “fitness score” to assess the individual response to diet, metabolism, and exercise
Source: J Int Soc Sports Nutr. 2022 Aug 2;19(1):455–73. doi: 10.1080/15502783.2022.2106148 (PMC9351578; doi:10.1080/15502783.2022.2106148)
Supplement: Supplemental Material [file RSSN_A_2106148_SM7975.pdf]

	Male	Female	Total
**n**	4	5	9
**Age [years]**	34.75 ± 4.03	22.42 ± 1.99	31.11 ± 5.42
**Age range [years]**	29 - 38	24 - 36	24 - 38
**BMI [T0, kg/m^2]**	25.30 ± 1.43	22.42 ± 1.99	23.70 ± 2.25
**BMI [T1, kg/m^2]**	25.03 ± 1.03	22.77 ± 2.32	23.77 ± 2.13
