# Supplementary material for: MiRNA-based “fitness score” to assess the individual response to diet, metabolism, and exercise
Source: J Int Soc Sports Nutr. 2022 Aug 2;19(1):455–73. doi: 10.1080/15502783.2022.2106148 (PMC9351578; doi:10.1080/15502783.2022.2106148)
Supplement: Supplemental Material [file RSSN_A_2106148_SM7974.pdf]

	Male	Female	Total
**n**	20	41	61
**Age [years]**	34.85 ± 6.47	31.73 ± 9.67	32.75 ± 8.82
**Age range [years]**	24 - 52	19 - 55	19 - 55
**BMI [T0, kg/m^2]**	24.86 ± 2.85	23.50 ± 3.19	23.95 ± 3.13
**BMI [T1, kg/m^2]**	24.79 ± 2.69	23.38 ± 2.99	23.84 ± 2.95
**Basal metabolic rate [T0, kcal]**	1667 ± 121	1336 ± 78	1444 ± 183
**Basal metabolic rate [T1, kcal]**	1687 ± 117	1360 ± 70	1468 ± 177
**Phase angel [T0, °]**	6.34 ± 0.54	5.77 ± 0.37	5.95 ± 0.54
**Phase angel [T1, °]**	6.41 ± 0.52	5.97 ± 0.50	6.11 ± 0.54
**LBM [T0, kg]**	62.08 ± 6.20	44.62 ± 4.88	50.34 ± 9.85
**LBM [T1, kg]**	62.92 ± 6.33	45.22 ± 4.62	51.02 ± 9.85
**BFM [T0, kg]**	18.81 ± 5.58	21.08 ± 6.29	20.34 ± 6.12
**BFM [T1, kg]**	17.48 ± 6.29	20.04 ± 6.12	19.20 ± 6.24
**BCM [T0, kg]**	33.23 ± 4.06	22.74 ± 2.42	26.18 ± 5.81
**BCM [T1, kg]**	33.87 ± 3.69	23.57 ± 2.22	26.95 ± 5.60
**ICW [T0, l]**	26.77 ± 1.96	20.28 ± 0.99	22.40 ± 3.36
**ICW [T1, l]**	27.10 ± 1.93	20.58 ± 0.92	22.72 ± 3.36
**ECW [T0, l]**	18.68 ± 2.60	12.59 ± 2.32	14.58 ± 3.75
**ECW [T1, l]**	18.97 ± 2.73	12.74 ± 2.26	14.78 ± 3.80
